# Supplementary material for: The effect of the erythropoietin resistance index on brain hemorrhage and infarction risk in maintenance hemodialysis patients: a retrospective cohort study
Source: PeerJ. 2025 Nov 12;13:e20326. doi: 10.7717/peerj.20326 (PMC12619578; doi:10.7717/peerj.20326)
Supplement: Supplemental Information 2 — The meaning corresponding to 1 and 2 in the data [file peerj-13-20326-s002.docx]

codebook

Type of primary kidney disease:1=Hypertention,2=Diabetes;3=Chronic glomerulonephritis;4=Others/unkown

Coronary heart disease:1=yes,0=no

Hypertention:1=yes,0=no

Diabetes:1=yes,0=no

Atrial fibrillation：1=yes,0=no

History of stroke:1=yes,0=no

Aspirin:1=yes,0=no

ACEI/ARB:1=yes,0=no

Iron supplementation:1=yes,0=no

brain hemorrhage:1=yes,0=no

brain infarction:1=yes,0=no

Death:1=yes,0=no

ERI group: 1=group 1 ;2=group 2 ; 3=group 3; 4=group 4.
