# Supplementary material for: The effect of the erythropoietin resistance index on brain hemorrhage and infarction risk in maintenance hemodialysis patients: a retrospective cohort study
Source: PeerJ. 2025 Nov 12;13:e20326. doi: 10.7717/peerj.20326 (PMC12619578; doi:10.7717/peerj.20326)
Supplement: Supplemental Information 3 [file peerj-13-20326-s003.docx]

STROBE Statement—checklist of items that should be included in reports of observational studies

|  | Item No. | Recommendation | Page  No. | Relevant text from manuscript |
| --- | --- | --- | --- | --- |
| **Title and abstract** | 1 | (*a*) Indicate the study’s design with a commonly used term in the title or the abstract | 1,2 | The effect of---in MHD patients. |
|  |  | (*b*) Provide in the abstract an informative and balanced summary of what was done and what was found | 1,2 | The effect of---in MHD patients. |
| Introduction | | | |  |
| Background/rationale | 2 | Explain the scientific background and rationale for the investigation being reported | 3 | The number of hemodialysis--patients are still unclear. |
| Objectives | 3 | State specific objectives, including any prespecified hypotheses | 3 | Our study was performed-- mortality in MHD patients |
| Methods | | | |  |
| Study design | 4 | Present key elements of study design early in the paper | 3,4 | In this retrospective study--with approval number Y2024--204. |
| Setting | 5 | Describe the setting, locations, and relevant dates, including periods of recruitment, exposure, follow-up, and data collection | 3,4,5 | In this retrospective study--according to the ERI quartile. |
| Participants | 6 | (*a*) *Cohort study*—Give the eligibility criteria, and the sources and methods of selection of participants. Describe methods of follow-up  *Case-control study*—Give the eligibility criteria, and the sources and methods of case ascertainment and control selection. Give the rationale for the choice of cases and controls  *Cross-sectional study*—Give the eligibility criteria, and the sources and methods of selection of participants | 3,4,5 | In this retrospective study--according to the ERI quartile. |
|  |  | (*b*) *Cohort study*—For matched studies, give matching criteria and number of exposed and unexposed  *Case-control study*—For matched studies, give matching criteria and the number of controls per case |  |  |
| Variables | 7 | Clearly define all outcomes, exposures, predictors, potential confounders, and effect modifiers. Give diagnostic criteria, if applicable | 3,4,5 | In this retrospective study--according to the ERI quartile. |
| Data sources/ measurement | 8* | For each variable of interest, give sources of data and details of methods of assessment (measurement). Describe comparability of assessment methods if there is more than one group | 3,4,5 | In this retrospective study--according to the ERI quartile. |
| Bias | 9 | Describe any efforts to address potential sources of bias | 5 | The measurement data are expressed-- p < 0.05 was considered statistically significant. |
| Study size | 10 | Explain how the study size was arrived at | 5 | The measurement data are expressed-- p < 0.05 was considered statistically significant. |

Continued on next page

| Quantitative variables | 11 | Explain how quantitative variables were handled in the analyses. If applicable, describe which groupings were chosen and why | 5 | The measurement data are expressed-- p < 0.05 was considered statistically significant. |
| --- | --- | --- | --- | --- |
| Statistical methods | 12 | (*a*) Describe all statistical methods, including those used to control for confounding | 5 | The measurement data are expressed-- p < 0.05 was considered statistically significant. |
|  |  | (*b*) Describe any methods used to examine subgroups and interactions | 5 | The measurement data are expressed-- p < 0.05 was considered statistically significant. |
|  |  | (*c*) Explain how missing data were addressed | 5 | The measurement data are expressed-- p < 0.05 was considered statistically significant. |
|  |  | (*d*) *Cohort study*—If applicable, explain how loss to follow-up was addressed  *Case-control study*—If applicable, explain how matching of cases and controls was addressed  *Cross-sectional study*—If applicable, describe analytical methods taking account of sampling strategy | 5 | The measurement data are expressed-- p < 0.05 was considered statistically significant. |
|  |  | (*e*) Describe any sensitivity analyses | 5 |  |
| Results | | | | |
| Participants | 13* | (a) Report numbers of individuals at each stage of study—eg numbers potentially eligible, examined for eligibility, confirmed eligible, included in the study, completing follow-up, and analysed | 6 | Data were collected from-- stratified by ERI quartile (Table 1). |
|  |  | (b) Give reasons for non-participation at each stage |  |  |
|  |  | (c) Consider use of a flow diagram |  |  |
| Descriptive data | 14* | (a) Give characteristics of study participants (eg demographic, clinical, social) and information on exposures and potential confounders | 6 | Data were collected from-- stratified by ERI quartile (Table 1). |
|  |  | (b) Indicate number of participants with missing data for each variable of interest | 6 | Data were collected from-- stratified by ERI quartile (Table 1). |
|  |  | (c) *Cohort study*—Summarise follow-up time (eg, average and total amount) | 6 | Data were collected from-- stratified by ERI quartile (Table 1). |
| Outcome data | 15* | *Cohort study*—Report numbers of outcome events or summary measures over time | *6* | Data were collected from-- stratified by ERI quartile (Table 1). |
|  |  | *Case-control study—*Report numbers in each exposure category, or summary measures of exposure |  |  |
|  |  | *Cross-sectional study—*Report numbers of outcome events or summary measures |  |  |
| Main results | 16 | (*a*) Give unadjusted estimates and, if applicable, confounder-adjusted estimates and their precision (eg, 95% confidence interval). Make clear which confounders were adjusted for and why they were included | 6 | The Kaplan‒Meier survival --all-cause mortality (HR: 1.72 95% CI: (1.05–2.82), p = 0.033). |
|  |  | (*b*) Report category boundaries when continuous variables were categorized |  |  |
|  |  | (*c*) If relevant, consider translating estimates of relative risk into absolute risk for a meaningful time period |  |  |

Continued on next page

| Other analyses | 17 | Report other analyses done—eg analyses of subgroups and interactions, and sensitivity analyses |  |  |
| --- | --- | --- | --- | --- |
| Discussion | | | | |
| Key results | 18 | Summarise key results with reference to study objectives | 7 | In this study, we found that a higher --and all-cause death in MHD patients. |
| Limitations | 19 | Discuss limitations of the study, taking into account sources of potential bias or imprecision. Discuss both direction and magnitude of any potential bias | 9 | This study has certain limitations--the effect of changes in the levels. |
| Interpretation | 20 | Give a cautious overall interpretation of results considering objectives, limitations, multiplicity of analyses, results from similar studies, and other relevant evidence | 7,8,9 | Patients on MHD have--masking the effect of changes in the levels. |
| Generalisability | 21 | Discuss the generalisability (external validity) of the study results |  |  |
| Other information | |  | | |
| Funding | 22 | Give the source of funding and the role of the funders for the present study and, if applicable, for the original study on which the present article is based | 10 | This study was supported by the--Joint Fund (2023-MSLH-350). |

*Give information separately for cases and controls in case-control studies and, if applicable, for exposed and unexposed groups in cohort and cross-sectional studies.

**Note:** An Explanation and Elaboration article discusses each checklist item and gives methodological background and published examples of transparent reporting. The STROBE checklist is best used in conjunction with this article (freely available on the Web sites of PLoS Medicine at http://www.plosmedicine.org/, Annals of Internal Medicine at http://www.annals.org/, and Epidemiology at http://www.epidem.com/). Information on the STROBE Initiative is available at www.strobe-statement.org.
